# Evolution of the scattering properties of phytoplankton cells from flow cytometry measurements

**DOI:** 10.1371/journal.pone.0181180

**Published:** 2017-07-14

**Authors:** William Moutier, Lucile Duforêt-Gaurier, Mélilotus Thyssen, Hubert Loisel, Xavier Mériaux, Lucie Courcot, David Dessailly, Anne-Hélène Rêve, Gérald Grégori, Séverine Alvain, Aude Barani, Laurent Brutier, Mathilde Dugenne

**Affiliations:** 1 Univ. Littoral Côte d’Opale, Univ. Lille, CNRS, UMR 8187, LOG, Laboratoire d’Océanologie et de Géosciences, F 62930 Wimereux, France; 2 Aix-Marseille Université, Université de Toulon, CNRS, IRD, MIO UM 110, Mediterranean Institute of Oceanography, 13288 Marseille, France; 3 Univ. Lille, UMR CNRS 8187 - LOG - Laboratoire d’Océanologie et de Géosciences, 62930 Wimereux, France; 4 Centre National de la Recherche Scientifique, UMR CNRS 8187 - LOG - Laboratoire d’Océanologie et de Géosciences, 62930 Wimereux, France; Ludwig-Maximilians-Universitat Munchen, GERMANY

## Abstract

After the exponential growth phase, variability in the scattering efficiency of phytoplankton cells over their complete life cycle is not well characterised. Bulk measurements are impacted by senescent cells and detritrus. Thus the analysis of the evolution of the optical properties thanks to their morphological and/or intra-cellular variations remains poorly studied. Using the Cytosense flow cytometer (CytoBuoy b.v., NL), the temporal course of the forward and sideward efficiencies of two phytoplankton species (*Thalassiosira pseudonana* and *Chlamydomonas concordia*) were analyzed during a complete life-cycle. These two species differ considerably from a morphological point of view. Over the whole experiment, the forward and sideward efficiencies of *Thalassiosira pseudonana* were, on average, respectively 2.2 and 1.6 times higher than the efficiencies of *Chlamydomonas concordia*. Large intra-species variability of the efficiencies were observed over the life cycle of the considered species. It highlights the importance of considering the optical properties of phytoplankton cells as a function of the population growth stage of the considered species. Furthermore, flow cytometry measurements were combined with radiative transfer simulations and biogeochemical and optical measurements. Results showed that the real refractive index of the chloroplast is a key parameter driving the sideward signal and that a simplistic two-layered model (cytoplasm-chloroplast) seems particularly appropriate to represent the phytoplankton cells.

## 1 Introduction

The scattering and absorption characteristics of ocean water and all its constituents are described by the Inherent Optical Properties (IOPs) [1, 2]. Over the last few decades, many studies have shown that the morphology and/or the internal structure of phytoplankton cells impact light absorption [3–5] and scattering [6–11]. For example, phytoplankton heterogeneity and inner complexity (gas vacuoles, chloroplast, silica wall, etc.) explain why the backscattering signal is higher than predicted by the Mie theory, which considers a particle (i.e., a phytoplankton cell) as an homogeneous sphere [7–10, 12–19, 19–24]. For instance, Dall’Olmo *et al*., 2009 showed that Mie simulations can match experimental measurements of backscattering if the real refractive index of particles used in simulations is around 1.09 which is higher than the average real refractive index expected for phytoplankton cell [25]. Bernard *et al*., 2009 [20] simulated the phytoplankton optical properties with a two layered sphere model. They highlighted that the real refractive index of the chloroplast and the relative volume of the chloroplast (*V*_chl_) are key parameters impacting the backward efficiency. However, while the morphology and the internal structure of cells change according to the cell growth and growth conditions (light, temperature, and nutrients concentrations) [5, 26–29], the phytoplankton optical propreties are considered as static and representative of the exponential growth phase. The intra- and inter-species variations of the scattered signal need to be investigate.

Over the last two decades, Hobilabs Hydroscat, WET Labs ECO-BB or ECO-VSF instruments were developed to perform routine *in situ* measurements of scattering at one or three scattering angles and then to assess the bulk backscattering coefficient. As bulk measurements are impacted by the various water constituents (e.g., phytoplankton, viruses and heterotrophic bacteria, detritus, sediments and air bubbles), they are therefore less sensitive to the optical variability occurring within a specific sub-population [30]. Consider the bulk information can constitute a limitation to analyse the optical properties of phytoplankton cells over a complet life cycle. In this context, flow cytometry is a valuable tool to analyze the individual scattering of particles, and move beyond the bulk information. The usefulness of the flow cytometry to improve our knowledge about the IOPs of individual marine cells has already been higlighted by different studies [10, 11, 30–36]. For instance, Ackleson *et al*., 1988 [31] developed a method to derive the refractive index and the particle size combining flow cytometer measurements and simulations from Mie theory. Ackleson *et al*., 1993 [33] observed short-term changes variations of the phytoplankton optical properties after a dilution of cell cultures with fresh media. These variations were associated to a rearrangement of the internal structure of the cells which induced a variations of the real part of the refractive index. The Cytosense benchtop flow cytometer (CytoBuoy b.v., NL) has been specifically designed to analyse the phytoplankton cells. The Cytosense flow cytometer measures the sideward (FSC; 2°-15°) and the forward (SSC; 45°-135°) scattering, as well as several fluorescence intensities of each single particle which passes through the focused 488 nm laser beam. Duforêt-Gaurier *et al*., 2015 developed a new methodology to derive the forward, sideward and backward cross sections of single particles from the measurements made by the Cytosense. A first validation of the method has been performed on bead suspensions [10], and then on phytoplankton cultures by Moutier *et al*., 2016 [11].

After this technical step of validation, we propose here to use the Cytosense to study the inter- and intra-species variations of phytoplankton cultures. The objectives of the present study is to identify which morphological or intra-cellular parameters induce inter- or intra-species variations of the scattering properties. For this purpose, the forward, sideward and backward cross sections were estimated, during a microcosm experiment, from Cytosense measurements using the Duforêt-Gaurier *et al*., 2015’s method. The experience was conducted on two different taxa (a flagellate green algae and a diatom) during a complete population growth cycle. These two species are distinct from a morphological point of view. The diatom *Thalassiosira pseudonana* stands for particles with a silica wall and single cells are in most cases cylindrical. The green algae *Chlamydomonas concordia* is a small ovoid and may potentially form aggregates during the experience. As the cell size, the shape, the thickness of the silica wall, the chlorophyll-*a* by cell or the aggregate configurations, change during the population growth cycle are expected. The originality of this study lies in combining cytometric measurements with biogeochemical measurements, Scanning Electron Microscopy (SEM) and radiative transfer simulations. Radiative transfer simulations are used to examine the impact of heterogeneity and aggregation to try to explain the variations observed on the different scattering parameters considered here. In this regards, the combination of in situ measurements and theoretical simulations has proven invaluable for assessing the cellular characteristics which impact the optical signal, as evidenced in previous studies [20, 23].

## 2 Material and method

### 2.1 Phytoplankton cultures

Cultures of *Thalassiosira pseudonana* (THAL) and *Chlamydomonas concordia* (CHLAM) were obtained from the Roscoff Culture Collection (RCC, http://roscoff-culturecollection.org). THAL and CHLAM were maintained in monospecific conditions in a f/2 medium at 17°C, under a photon flux density of about 100 *μ*mol.photons.m^-2^.s^-1^ with a 12:12h light:dark cycle. They were grown in batch mode during 15 days to obtain 4.5 L of culture. One day before the beginning of the microcosm experiment, the 4.5 L of culture were added to 60 L of fresh f/2 medium. A volume of 60 L is required to realize the daily sampling for biogeochemical analysis, and to maintain a sufficient depth (≥ 25 cm) for the optical measurements. THAL and CHLAM batches were duplicated; and named B1 and B2 for THAL and B4 and B5 for CHLAM. During the experiment, the cultures were maintained under a surface illumination of 200 *μ*mol.photons.m^-2^.s^-1^. The 12:12h light:dark cycle and the temperature of 17°C were maintained. Continuous agitation was applied to homogenize the culture and to avoid chain formation. The salinity of the four batches was between 29.1–29.7. To cover all the culture phases. the microcosm experiment was conducted over 20 days.

### 2.2 Flow cytometry

In this study, we used the Cytosense acquired in 2010 by the PRECYM flow cytometry platform of the Mediterranean Institute of Oceanography (MIO; http://precym.mio.univ-amu.fr). The Cytosense is a benchtop pulse-shape flow cytometer designed for the observation of phytoplankton cells. It counts and analyses particles with diameters between 1 and 800 *μ*m. The sample intake speed is 3 *μ*l.s^−1^. The particle suspension is injected into a free carrying sheath fluid, that narrows down the suspension into a thin line of fluid. The particles are aligned and separated along the path of the fluid in the flow cell (hydrodynamic focusing), which is perpendicular to the direction of the laser beam. Considering the design of the Cytosense and the analysis conditions, we assume that for non-spherical particles, the particle longer axis is vertically oriented perpendicularly to the laser beam. The velocity of the particle suspension is governed by the sheath flow rate, factory set at about 2.2 m.s^-1^. Particles flow one by one, through a 488 nm focused laser beam. The Cytosense measures the signal scattered by the particles in the forward and sideward direction. The fluorescence emitted by the photosynthetic pigments in algal cells is detected at three different spectral bandwidths. The resulting signatures are displayed as red (chlorophyll-*a* related), orange (phycoerythrin related) and yellow fluorescence intensities respectively, which assist in determining the pigment type and content of each particle. The Cytosense instrument uses PIN photodiodes to detect the forward scatter (FSC) and photomultiplier tubes to detect the right angle side scatter and fluorescence signals. The collection solid angle of the forward scatter detectors starts at ca. 2°, going up to ca. 15°. The sideward scattered light, together with the fluorescence emission, is collected at 90° relative to the laser beam (with a solid angle between 45° and 135°). Digital data acquisition is initiated when the particle enters into the laser beam and is terminated when the particle is no longer detected. The data recording is done at a frequency of 4MHz. Data recording was triggered by the SSC signal (15 mV). This trigger allows the removal of a part of the instrumental electronical noise and the signals from the smallest particles (such as debris, heterotrophic bacteria).

The proprietary CytoUSB software (CytoBuoy b.v., NL) was used to drive the instrument and collect the data. The proprietary Cytoclus software (CytoBuoy b.v., NL) was used to manually analyze the data collected by the Cytosense [37]. It enables the display and clustering of data points representing particles (cells) with similar optical properties, based on their forward and sideward scattering, and their fluorescence intensities. The clustering may use up to ten simple mathematical signal descriptors for each available detector signal (for example, length, height, centre of gravity, asymmetry, number of humps…). The various clusters are defined manually by drawing regions in correlated bivariate scatter plots. This combines objective factors from the cytograms and subjective considerations linked to the expertise of the observer. Moreover, an “image-in-flow” device, mounted in the Cytosense, takes pictures of the cells within a predefined region of interest.

### 2.3 Ancillary measurements

Every day, one or two samples per batch were collected and filtered through GF/F filters pre-combusted at 450°C (Whatman, diameter 25 mm, nominal pore size 0.7 microns). The samples were used to quantify, every day, the particulate absorption coefficient and the non-algal particles absorption coefficient (*a*_p_ and *a*_nap_, respectively) and, every two days, the concentrations of chlorophyll-a (Chl-*a*). After liquid nitrogen deep freezing, samples dedicated to Chl-*a* *a*_p_ and *a*_nap_ were stored at -80°C until analysis. The *a*_p_ and *a*_nap_ coefficients were quantified by spectrophotometry following the Tassan *et al*., 1995 and 2002 protocols [38, 39]. Chl-*a* was measured by fluorimetry [40]. The chlorophyll-*a* was subsequently extracted by grinding the filters in 4 ml of 90% acetone. Then, 4ml of 90% acetone were added and samples were stored in the dark at 4°C overnight. This was followed by centrifugation (for 15 minutes at 3,000 revolutions per min), and Chl-*a* was quantified in the supernatant by a Turner Designs fluorimeter. The fluorescence values were converted into Chl-*a* concentration using a standard Chl-*a* solution (Anacystis nidulans, Sigma).

A measure of *a*_p_ was made from 400 to 750 nm at a 1 nm bandwidth on a Shimadzu UV 2450 spectrophotometer equipped with an integrating sphere. Then, on the same filter, after a pigment extraction using NaClO 1% active chloride, another measurement was performed to obtain *a*_nap_. The pathlength amplification was corrected following the Tassan *et al*., 2002 [39] instructions. Phytoplankton absorption (*a*_phy_) was then obtained by subtracting of *a*_nap_ from *a*_p_.

### 2.4 Scanning Electron Microscope (SEM)

For both species, four samples were dedicated to SEM analysis. They correspond to the day 5, 11, 13 and 15 for THAL and the day 5, 11 and 15 for CHLAM (to account for different population growth stages). Samples were pre-treated immediately after collection by fixing them in lugol/glutaraldehyde 2.5% solution. For CHLAM, approximately 4 mL of culture were filtered through polycarbonate membrane (Millipore, 0.4 *μ*m) covered with polylisine solution. The filter obtained was then immediately put in a bath of 70% alcohol. Then, the sample was dehydrated through graded alcohol series up to 100% ethanol (Merck PA); subsequently, the filter was immersed in HMDS (Hexamethyl disilazane, Molekula) for 1/2 hour (twice). After, removing the excess of HMDS, sample was evaporated overnight under a fume hood. For THAL, approximately 1 mL of culture was filtered through poly-carbonate membrane (Millipore, 0.4 *μ*m). Subsequently, the filter was immersed in HCL 37% (Merck, PA) at room temperature during 24h. Then the acid solution was filtered and rinsed with MilliQ-water. Filters were then dried for 24 hours under a laminar flow hood.

Finally, all samples were mounted on aluminum stubs (Agar Scientific) with double sticky carbon tabs (Agar Scientific) and sputter coated under Argon flow with Au/Pd Polaron SC 7620 during 90s. Afterward the specimens were examined with a Scanning Electron Microscope (SEM LEO 438VP, accelerating voltage of 15 keV with a beam current of 10 pA)

### 2.5 Methodology

The methodology developed by Duforêt-Gaurier *et al*., 2015 was used to derive, from the Cytosense, the forward (*σ*_FSC_), sideward (*σ*_SSC_) and backward (*σ*_b_b__) cross sections (*μ*m^2^). First, the weighting functions for the forward and sideward detectors were calculated to convert numerical counts into cross sections (*μ*m^2^). Noting that very recently the weighting functions have been slightly modified for a better consideration of the optical system (Duforêt-Gaurier *et al*., 2016, personal communication). Then, for each particle, the *σ*_SSC_ was converted into *σ*_b_b__ using the theoretical relationship from Duforêt-Gaurier *et al*., 2015 and slightly modified by Moutier et al., 2016 [11]:

$$\begin{array}{c} \sigma_{{\text{b}}_{\text{b}}}\left(488\right)=10^{-0.6}\times {\sigma_{\text{SSC}}\left(488\right)}^{1.09}\;\;\;\;\left(R^{2}=0.9\right) \end{array}$$ (1)

The latter was established from the theoretical database including 590,000 simulations from the ScattnLay code [41, 42]. This database includes homogeneous, two and three layered spheres to represent the optical properties of a large diversity of phytoplankton cells (see details in [10] and [11]).

### 2.6 Algorithm

In this study, two open access radiative transfer codes (the ScattnLay; [41, 42] and the Generalized Multiparticle Mie-solution GMM; [43]) were used for computing the volume scattering function (VSF) of homogeneous spheres, layered spheres and aggregates. Please note that theses codes are available in free access. The forward and sideward signals were obtained by integrating the VSF over 2°-15° and 45°-135°, respectively. Radiative transfer computations were carried out given the wavelength of the incident radiation equal to 488 nm and the refractive index of water equal to 1.334 [44].

First, the ScattnLay code was used to simulate the optical properties of two and three layered sphere models. The two layered sphere models are composed of an inner layer, the cytoplasm (cyt) and, an outer layer, the chloroplast (chl). An exhaustive review of the internal structure of phytoplankton cells was performed by Bernard *et al*., 2009 [20]. We used this review to define the relevant refractive indices and the relative proportions of the different layers.

Cells, as THAL, compose of a silica wall (Si) are simulated as three layered spheres. The relative volumes of the silica wall (*V*_Si_) were measured from SEM micrographs (see section 3.3). To analyze the influence of the evolution of the silica wall thickness, we arbitrarily chose the 4^*th*^ and the 13^*th*^ days of the experiment. The relative proportions are: 70%_cyt_-30%_chl_, 80%_cyt_-20%_chl_, 80%_cyt_-18.625%_chl_-1.375%_Si_ and 80%_cyt_-18.18%_chl_-1.82%_Si_. The description and the code inputs were detailed in [11]. Briefly, the complex refractive indices of the cytoplasm (*m*(cyt)) and the silica wall (*m*(Si)) were respectively fixed to: 1.02 + *i*2.0739 × 10^−4^ and 1.07 + *i*0.0001. Concerning the chloroplast, *m*_*r*_(*chl*) and *m*_*i*_(*chl*) are calculated according to the Gladstone and Dale formula [45]:

$$\begin{array}{c} \sum_{j}\;m_{\text{j}}\nu_{\text{j}}=m, \end{array}$$ (2)

where *m*_j_ and *ν*_j_ are the complex refractive index and the relative volume of the j-th layer, and *m* is the complex equivalent refractive index (*m* = *m*_r_ + *im*_i_) of the whole particle. The knowledge of the complex equivalent refractive index is useful to compare the simulations of heterogeneous spheres among themselves, regardless the number of layers and the relative proportion of each layer.

For each model, simulations were performed for a cell diameter between 1 to 40 *μ*m (0.166 *μ*m increment). The number of cases used in this study is about 1,000.

**Fig 1:**
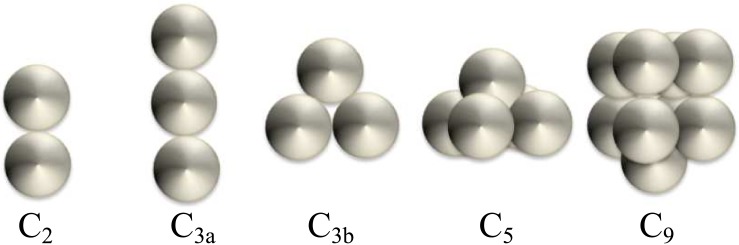
Geometry of the simulated aggregates. C stands for clusters, the number indicates the number of spheres and the letter in small cap stands for the various configurations.

The Generalized Multiparticle Mie-solution code (GMM; [43]) was used to simulate the light interaction with aggregates (details in [11]). Computations were performed for 5 different cell clusters (Fig 1). They were composed of 2, 3, 5 and 9 spheres corresponding to the most frequently encountered configurations as observed by SEM. For each sphere composing the clusters, the diameter is of 4 *μ*m and the refractive index is of *m* = 1.05 + *i*0.01.

## 3 Results

### 3.1 Flow cytometry analyses

Every morning, the Cytosense carried out single particle (cell) analyses for the four batches, over 20 days. From each Cytosense analysis, living phytoplankton cells were identified (see section 2.2). A distinction was also made between clusters corresponding to living cells of THAL and clusters corresponding to living cells of CHLAM to detect any possible contamination. The forward (FSC) and sideward scatter signals (SSC) were converted into forward (*σ*_FSC_) and sideward (*σ*_SSC_) scattering cross sections according to the Duforêt-Gaurier *et al*., 2015’s methodology (see section 2.5). In addition, the Cytosense provided an estimation of the particle longer axis thanks to the pulse shapes recorded by the instrument. In the following, the longer axis is assumed to be equal to the diameter (see details in [11]). For CHLAM and THAL, about one per cent of the data corresponded to particles with a diameter between 40 and 100 *μ*m. These values were quite high compared with diameters measured from optical or SEM microscopy and also with diameters referenced in the literature for the two species [16, 21, 46]. Outliers were excluded by a cut-off value applied at the last percentile (q = 0.99) to obtain a population representative of each species. Then, for each cluster, we calculated the median value for the *σ*_FSC_, *σ*_SSC_ and cell diameter named ${\tilde{\sigma }}_{\text{FSC}}$, ${\tilde{\sigma }}_{\text{SSC}}$ and $\tilde{D}$, respectively. The median value was preferred to the mean value as the probability density functions (PDF) of cross sections did not follow a Gaussian distribution. The median values are close to the PDF maximum, whereas mean values are much higher. For each measurement, forward, sideward and backward efficiency factor $\tilde{Q}$ has been calculated as following [44]:

$$\begin{array}{c} {\tilde{Q}}_{\text{FSC}/\text{SSC}/{\text{b}}_{\text{b}}}=\frac{4{\tilde{\sigma }}_{\text{FSC}/\text{SSC}/{\text{b}}_{\text{b}}}}{\pi {\tilde{D}}^{2}}. \end{array}$$ (3)

### 3.2 Abundance

**Fig 2:**
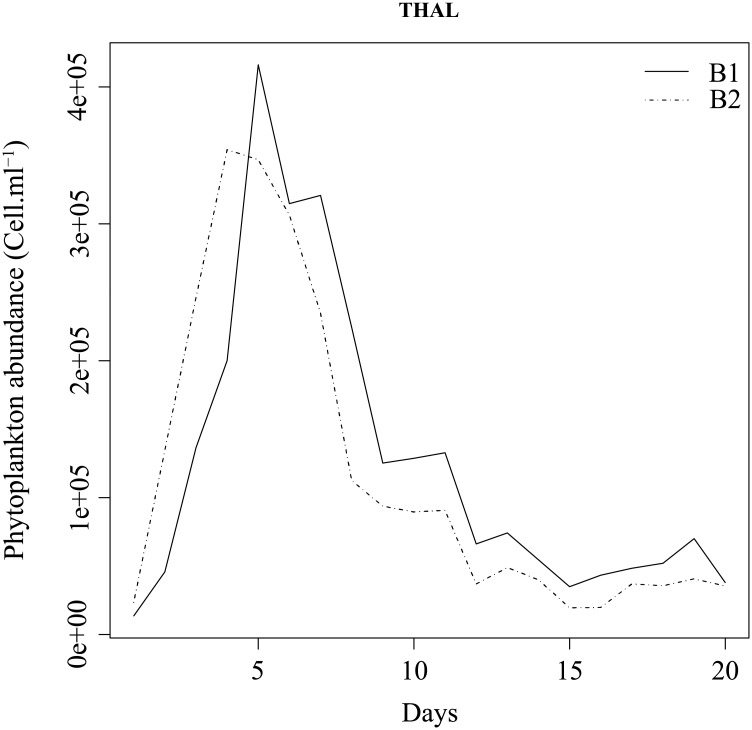
Abundance of phytoplanktonic cells over the time of experiment for B1 (solid line) and B2 (dashed line).

**Fig 3:**
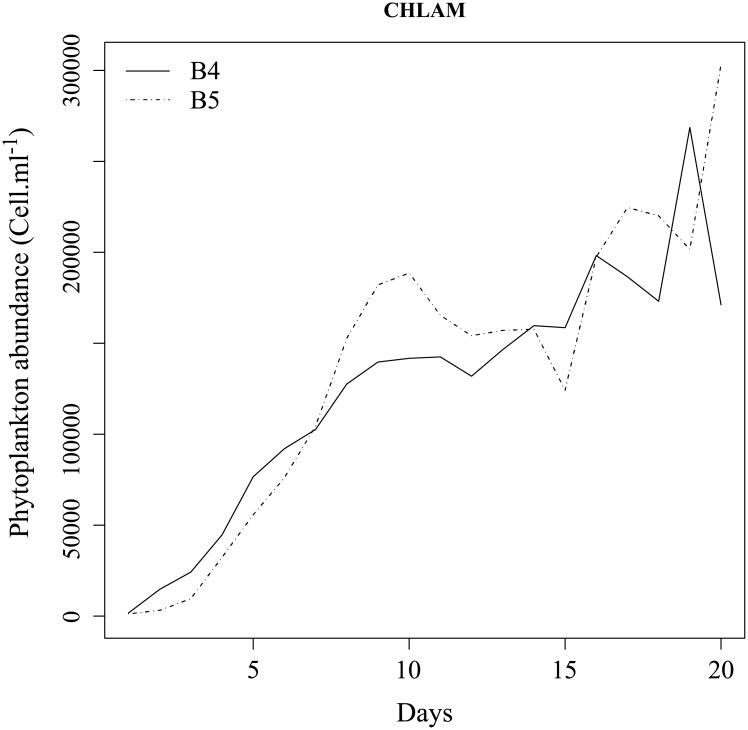
Abundance of phytoplanktonic cells over the time of experiment for B4 (solid line) and B5 (dashed line).

The Figs 2 and 3 show the abundance of living phytoplankton cells for THAL and CHLAM, respectively. For THAL (B1 and B2), the exponential growth phase is characterized by an increase of living phytoplankton cells from day 1 to 5. The end of the exponential phase is probably caused by a nutrient limitation. The senescence phase (day 6 to day 12) begins with the decrease of living phytoplankton cells. Then, the abundance of phytoplankton cells remains relatively constant from day 13 to day 20. For CHLAM (B3 and B4), the exponential phase is from the day 1 to 9. As THAL, the end of the exponential phase is probably due to a nutrient limitation. During the stationary phase (from day 10 to 15), the phytoplankton abundance remains rather constant. Contrary to THAL, there is no senescent phase. Indeed, from the day 16 to 20, the phytoplankton abundance increases. The increase in phytoplankton cells could be due to a dislocation of some aggregates or nutrient remineralization due to the action of bacterial activity. Through bacterial lysis, organic matter and nutrients are released into the medium available for heterotrophic prokaryotes and phytoplankton growth [47–50].

### 3.3 Morphology of the phytoplankton species

**Fig 4:**
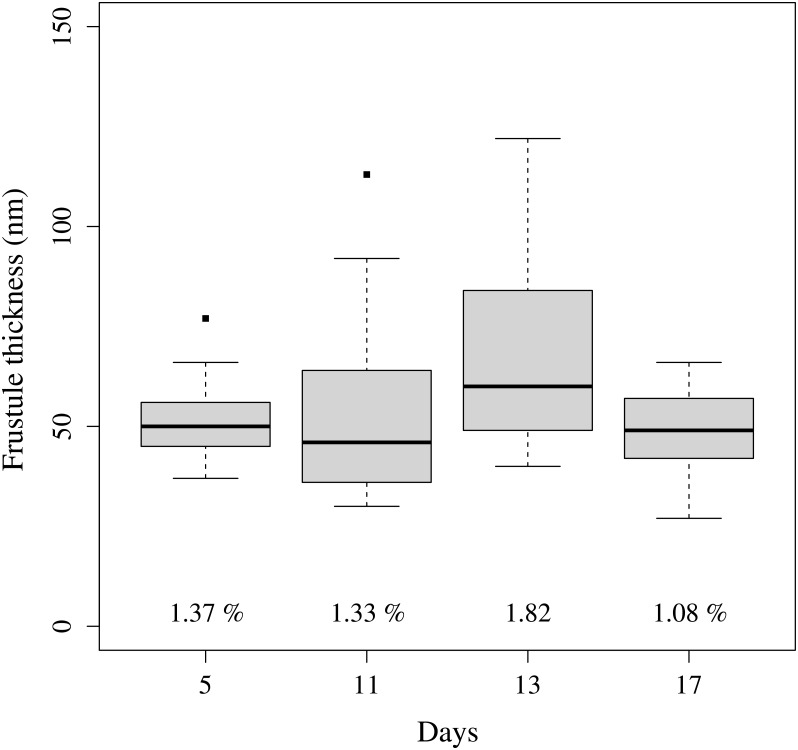
Boxplot of the mean frustule thickness for the 5^*th*^, 11^*th*^, 13^*th*^ and 17^*th*^ day of the experiment. The numbers under the boxplot represents the average proportion (%) of the frustule with respect to the cell diameter. Boxes extend from the 25^*th*^ to the 75^*th*^ percentile and the line indicate the median value. Values higher than 1.5 times the length of the box are considered as outliers and indicated by squares.

THAL is a marine centric diatom and represents particles with a silica wall. Images obtained from Scanning Electron Microscope (SEM), for days 5, 11, 13 and 17, showed that the shape of single cells was cylindrical with a mean surface-equivalent diameter (*D*_*e*_) between 5 and 7 *μ*m. Noted that from day 9 to the end of the experiment, aggregates, appearing as chains, were observed by SEM images and Cytosense analyses. Each single cell forming aggregate is displayed as a “hump” on the pulse shape profile of the entire particle (aggregate) recorded by the Cytosense. On the basis of the cytograms and particle optical profiles, a particle length of 13 *μ*m is considered as the size limit between single and aggregate cells. From day 1 to 9, between 4 and 15% of the cells were identified as aggregates, while between 20% and 40% of the cells were identified as aggregates for the rest of the experiment. Fig 4 displays the variations of the frustule thickness as observed for day 5, 11, 13, and 17.

CHLAM is a flagellate green algae which, contrary to THAL, does not have a silica wall. SEM images showed that the morphology of the CHLAM cells in culture can appear as a small single ovoid with a flagellum or an aggregate appearing as a group of cells. From SEM micrographs, we observed on average about 43% of aggregates and 57% of single cells throughout the whole experiment. Studying, how cells are arranged to form the aggregate are not evidenced on cytograms. Contrary to THAL where the cells are connected one after the other. Thus, the identification of each single sphere, and so of aggregates, from the recorded particle (aggregate) profiles was not possible. During the experiment, the mean *D*_*e*_ was between 2 and 34 *μ*m

### 3.4 The optical properties of species

#### 3.4.1 The temporal course

**Fig 5:**
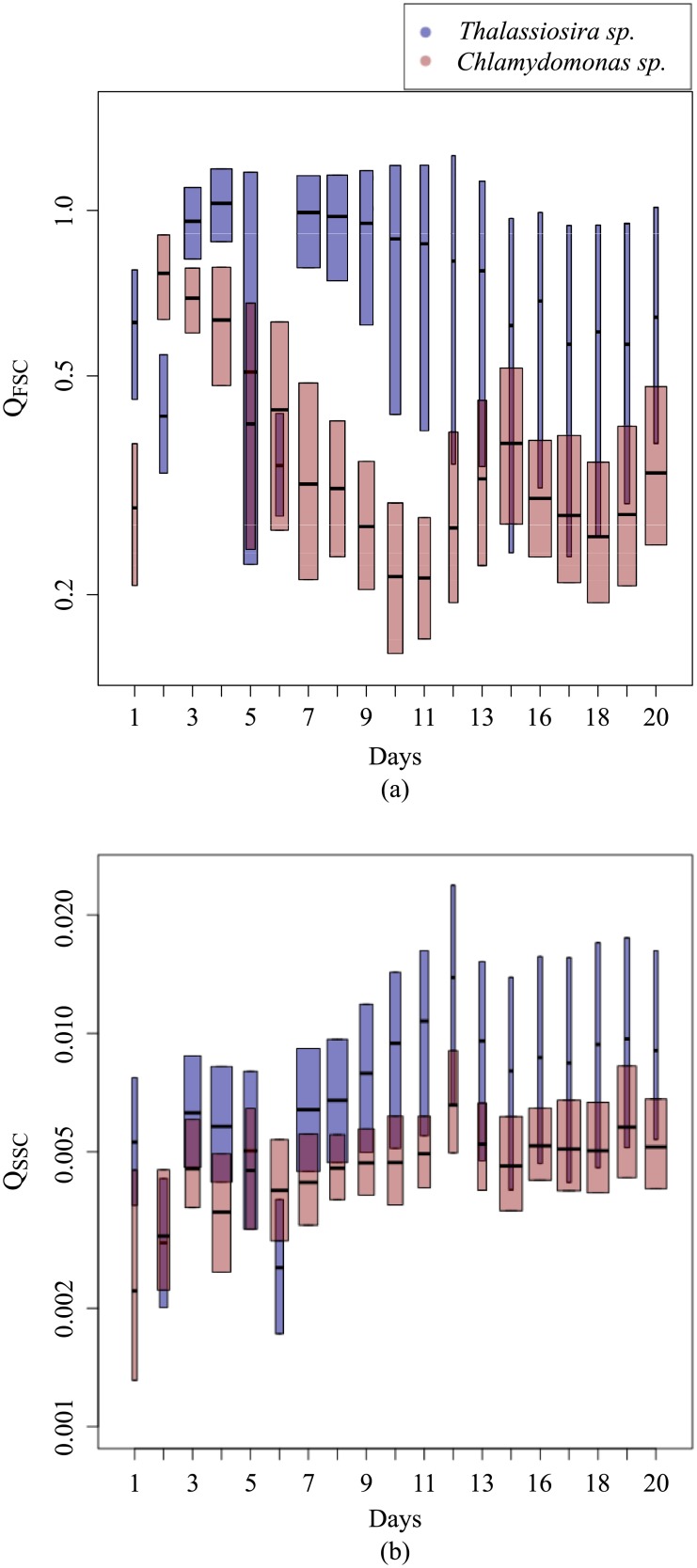
Temporal course of the (a) forward and (b) sideward efficiencies (log-linear scale) of THAL (blue box) and CHLAM (red box). The box width is proportional to the square-roots of the sample size.

**Fig 6:**
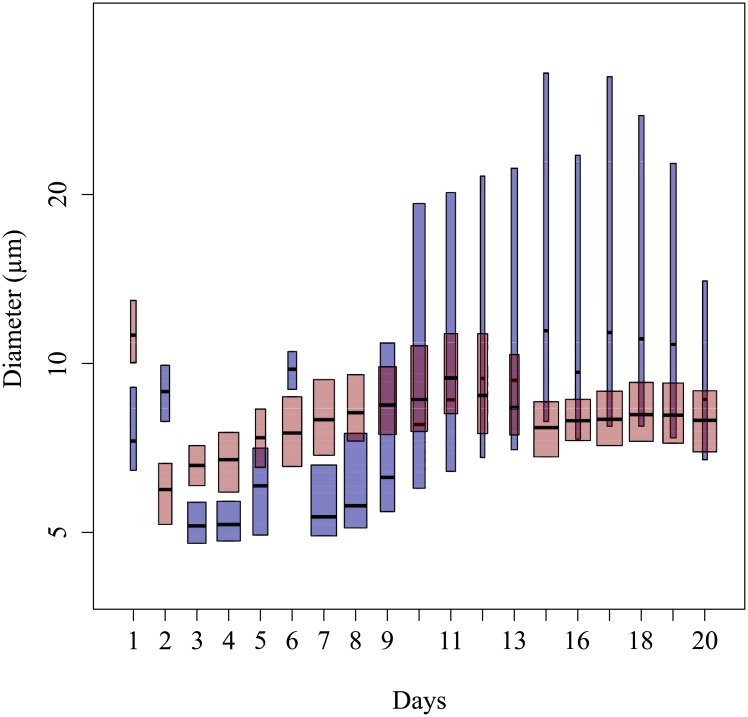
Same as Fig 5 but for the diameter.

As the study is dedicated to the impact of cell heterogeneity on the scattering signal, the efficiency factor is presented instead of the cross section because it is less influenced by the cell size (Fig 5). Indeed, the efficiency factor depends on the cell size relative to the wavelength, on the particle shape and on the distribution of the complex refractive index inside the particle [51]. In contrast, the effect of cell size on the cross section is twofold: directly by the geometrical cross section (Eq (3)) and by the efficiency factor [52, 53]. It is worth noticing that the present study was focused on living phytoplankton cells. The forward (*Q*_FSC_) and sideward (*Q*_SSC_) efficiencies were calculated, for each cell, according to Eq (3) using the cell diameter as provided by the Cytosense. As for the cross section, the median values (${\tilde{Q}}_{\text{FSC}}$ and ${\tilde{Q}}_{\text{SSC}}$) were calculated for each day of the experiment. The mean values of ${\tilde{Q}}_{\text{FSC}}$ and ${\tilde{Q}}_{\text{SSC}}$ were computed over the time period of the experiment with their associated coefficients of variation (%CV = 100 × standard deviation/mean). For both THAL and CHLAM, the mean values of ${\tilde{Q}}_{\text{FSC}}$ and ${\tilde{Q}}_{\text{SSC}}$ and their respective temporal courses were rather similar between the duplicates. Consequently, results are only presented for B1 and B4. For THAL, $<{\tilde{Q}}_{\text{FSC}}>$ was equal to 7.3 × 10^−1^ with CV of 28% and $<{\tilde{Q}}_{\text{SSC}}>$ was of 7.7 × 10^−3^ (CV = 36%). For CHLAM, $<{\tilde{Q}}_{\text{FSC}}>$ was around 0.36 (CV = 43%) and $<{\tilde{Q}}_{\text{SSC}}>$ was about 4.5 × 10^−3^ (CV = 21%). Diameter variations were observed, with the Cytosense, over the course of the experiment. For B1, the diameter $\tilde{D}$ varied between 5.14 and 11.45 *μ*m with $<\tilde{D}>$ = 8.29 *μ*m (CV = 26%). Concerning B4, $\tilde{D}$ varied between 5.97 and 11.23 *μ*m with $<\tilde{D}>$ of 7.97 *μ*m (CV = 13%) (Fig 6). Note that the discrepancies between the size estimated by the cytometer and observed by SEM microscopy (section 3.3) were already discussed in [11]. Over the whole experiment, the forward and sideward scattering efficiencies were, respectively, about 2.2 and 1.6 times higher for THAL than for CHLAM. The temporal course of ${\tilde{Q}}_{\text{FSC}}$ and ${\tilde{Q}}_{\text{SSC}}$ was different between the two species. For THAL, no specific trend was observed for ${\tilde{Q}}_{\text{FSC}}$ from day 1 to day 7. Then, ${\tilde{Q}}_{\text{FSC}}$ decreased from day 7 to day 15 and remained relatively constant from day 15 to the end of the experiment. For CHLAM, ${\tilde{Q}}_{\text{FSC}}$ decreased from day 2 to day 11 and from day 15 to day 18; it increased from day 11 to day 15 and from day 18 to day 20. The time variations of ${\tilde{Q}}_{\text{SSC}}$ for THAL and CHLAM were quite similar. No trend was observed from days 1 to 6. Then, the values increased from days 6 to 12. A decrease was observed from days 12 to 15 followed by a slight increase until the end of the experiment. For THAL, the variability of the scattering efficiencies increased with time as indicated by the length of the boxplots.

#### 3.4.2 Principal component analysis

**Fig 7:**
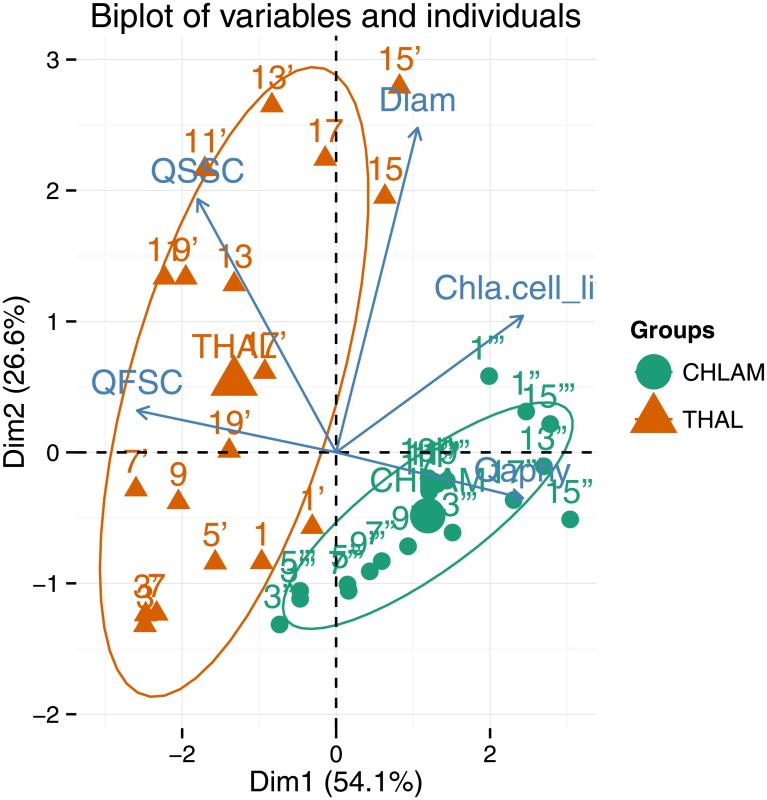
Principal component analysis (PCA) performed on the dataset. Projection of the observations (colored points) for all the available dates and the variables (arrows) on the two first principal components (80.7% of the total variance).

A Principal Component Analysis (PCA; R software 3.2.2) was used to compare the repartitions of the two species as a function of the forward and sideward efficiencies, the Chl-*a* concentration per living cell ($\text{Chl-}a.{\text{cell}}_{\text{liv}}^{-1}$; mg.m^−3^), the phytoplanktonic absorption efficiency factor (*Q*_*a*_phy__) and the cell diameter (Fig 7). This analysis was carried out to identify which variables influence the scattering of phytoplankton cells. Fig 7 displays only data with coincident cytometric, a_phy_ and Chl-*a* measurements.

The variance of our data is explained at 80.7% with the two first principal components (axes). The first principal component explains 54.1% of the variance, whereas the second one explains 26.6%. The forward efficiency (29.3%) contributed negatively to axis 1 with a cos^2^ of 0.8, while the $\text{Chl-}a.{\text{cell}}_{\text{liv}}^{-1}$ (25.8%) and *Q*_*a*_phy__ (25.81%) contributed positively to axis 1 with a cos^2^ of 0.7 and 0.7 respectively. The diameter (56.9%) and the sideward efficiency (33.4%) contributed positively to axis 2 with a cos^2^ of 0.7 and 0.4 respectively. The second axis opposes the THAL group (orange upward-pointing triangles) to the CHLAM group (green dots). CHLAM was characterized by lower scattering efficiencies and higher Chl-*a* contents per cell. The $\text{Chl-}a.{\text{cell}}_{\text{liv}}^{-1}$ for CHLAM was on average 4.8 times higher than for THAL. The mean $\text{Chl-}a.{\text{cell}}_{\text{liv}}^{-9}$ for THAL and CHLAM were, respectively, of 3.5 × 10^−9^ (CV = 108%) and of 6.8 × 10^−9^ (CV = 50%). THAL data are more scattered than CHLAM data. It is due to the high variability in $\text{Chl-}a.{\text{cell}}_{\text{liv}}^{-1}$ but also in ${\tilde{Q}}_{\text{SSC}}$, ${\tilde{Q}}_{\text{FSC}}$ and $\tilde{D}$. The variability and the differences between THAL and CHLAM may be explained by morphological and intra-cellular differences: (i) the higher structural complexity of diatoms (presence of a frustule and vacuole(s)); (ii) the aggregates, and (iii) the size difference. In the following paragraphs, we will examine if these hypotheses are consistent with ancillary measurements and the theoretical computations of radiative transfer codes.

#### 3.4.3 Influence of the $\text{Chl-}a.{\text{cell}}_{\text{liv}}^{-1}$ and the cell structure on *Q*_FSC_ and *Q*_SSC_

Our *in vitro* measurements indicate that the $\text{Chl-}a.{\text{cell}}_{\text{liv}}^{-1}$ is smaller for THAL. The lower Chl-*a* concentration of diatoms is mainly due to their specific cellular structure. Pigment concentration is influenced by the presence of a cell vacuole which restricts the chloroplasts to a thin layer [54] and reference therein). Janssen *et al*., 2001 [55] and Rosen *et al*., 1984 [27] showed that a reduction of the relative volume of the chloroplast (V_Chl_) is induced by an increase of the relative volume of the vacuole. This is consistent with Bernard *et al*., 2009, who referenced a mean *V*_Chl_ of 17.1% for diatoms, whereas they referenced a *V*_Chl_ of 52% for Chlorophytes.

A smaller *V*_Chl_ may induce more densely packed pigments where the chlorophyll molecules shade each other and the probability for a photon to be caught decreases. The consequence of this package effect is that Q_a_ decreases with an increase of the pigment density [56]. The mean *Q*_*a*_phy__, calculated over the whole experiment, was of 0.43 (CV = 63%, n = 37) for THAL, whereas it was of 1.09 (CV = 56%, n = 38) for CHLAM. This is consistent with Mas *et al*., 2008 [57] who found a higher package effect for diatoms. A lower $\text{Chl-}a.{\text{cell}}_{\text{liv}}^{-1}$ and the package effect induce a smaller imaginary refractive index, which is consistent with Stramski *et al*., 1999 [58].

**Fig 8:**
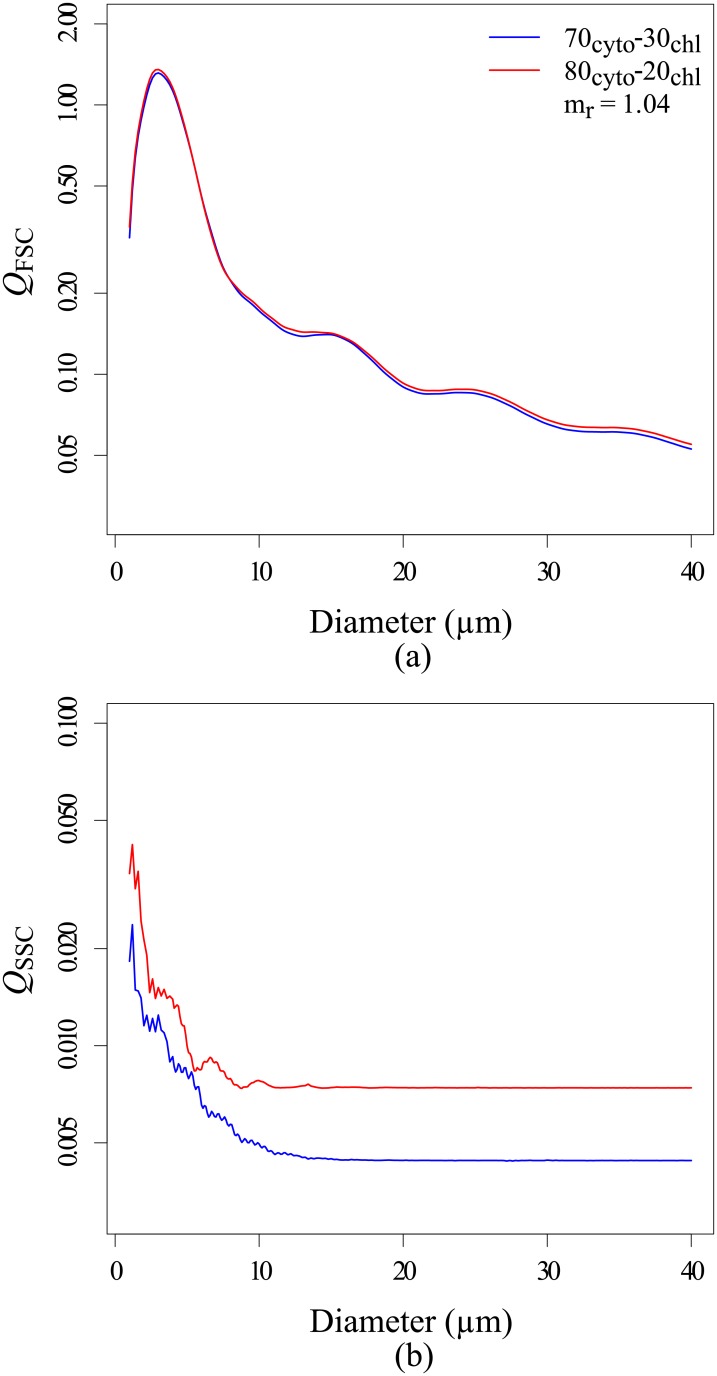
Forward (a) and sideward (b) efficiencies against cell diameter for the two models: 70%_cyt_-30%_chl_ (blue lines) and 80%_cyt_-20%_chl_ (red lines), for a volume equivalent complex refractive index (*m*) equal to: 1.04 + *i*0.01.

From simulations of the optical properties of multi-layered spheres, Bernard *et al*., 2009 showed that the impact of the imaginary refractive index of the chloroplast is weak on the total and backward scattering efficiencies. In this work, similar observations were done on the forward and sideward efficiencies (results not shown). If the pigments are more densely packed, the chloroplast is denser, its refringence and thus its real refractive index is higher [45]. Bernard *et al*., 2009 [20] highlighted that a higher real refractive index for the chloroplast leads to a higher total and backward efficiency. As Bernard *et al*., 2009’s observations deals with the total and backward scattering, they cannot be directly used to explain the variations of the forward and sideward efficiencies. So, additional radiative transfer computations were conducted. Two-layered sphere models were considered: 70%_cyt_-30%_chl_ and 80%_cyt_-20%_chl_. Fig 8 displays the Q_FSC_ and the Q_SSC_ against the cell diameter considering an equivalent complex refractive index (*m*) of 1.04 + *i*0.01. The corresponding complex refractive indices of the chloroplast are given in Table 1. For Q_FSC_, similar values are observed regardless to the model or the *m*_r_(chl). At the opposite, Q_SSC_ values vary with the considered model. Q_SSC_ values increase of 58% when the *m*_r_(chl) increase from 1.09 (70%_cyt_-30%_chl_) to 1.12 (80%_cyt_-20%_chl_). Same observations were done for other volume equivalent refractive indices (results not shown). Thus, as concluded by Bernard *et al*., 2009 on backscattering signal, the real refractive index of the chloroplast *m*_r_(chl) is a key parameter controlling the Q_SSC_. To summarize, variations of *V*(chl), which induce variations of *m*_r_(chl), can contribute to the inter- and intra-species variations of Q_SSC_ observed during the experiment. However, they do not explain Q_FSC_ differences as no change is observed from all the simulated study cases.

**Table 1:** Complex refractive index of the chloroplast and the volume equivalent complex refractive index for the 70%_cyt_-30%_chl_ and the 80%_cyt_-20%_chl_ models.

		70%_cyt_-30%_chl_	80%_cyt_-20%_chl_
Chl^1^	*m*_r_(chl)	1.0867	1.12
	*m*_i_(chl)	0.0333	0.0499
EVS^2^	*m*_r_	1.04	1.04
	*m*_i_	0.01	0.01

^1^ Chloroplast

^2^ Equivalent refractive index

#### 3.4.4 Influence of aggregates

**Fig 9:**
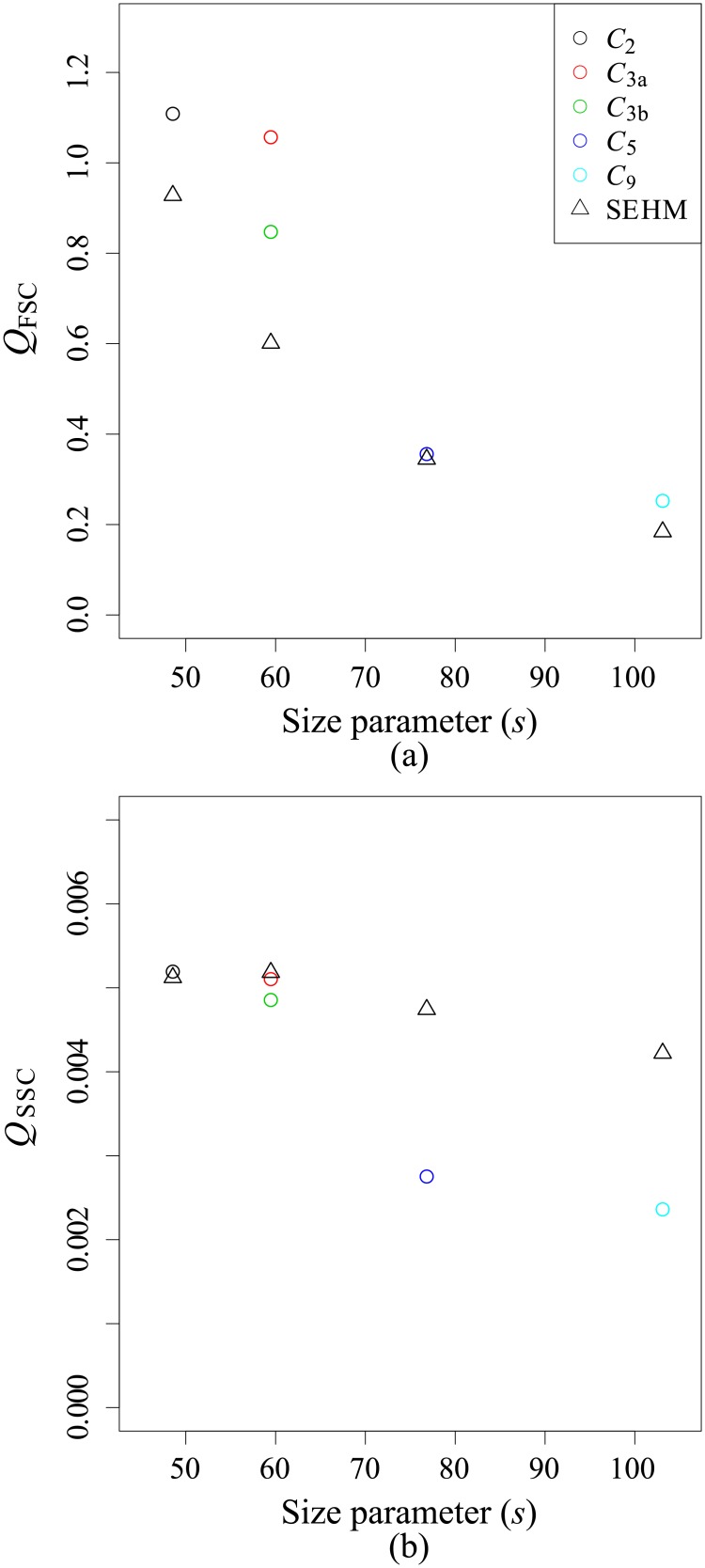
(a) The forward and (b) sideward efficiencies for aggregates (empty circle) and their surface equivalent homogeneous sphere (SEHS; empty triangle) as a function of the size parameter (*s*).

For CHLAM, the occurrence of aggregates was identified over the whole experiment, whereas for THAL, the aggregates were detected only during the senescence phase. Boss *et al*., 2009 [59] showed an impact of aggregates on light attenuation and Hatcher *et al*., 2001 [60] on the backscattering coefficient. In this study, radiative transfer computations were carried out to study the impact of aggregates on scattering efficiencies. Fig 9 displays *Q*_FSC_ and *Q*_SSC_ for five various aggregates composed of homogeneous spheres (Fig 1). Aggregates composed of multi-layered spheres or spheroids are not simulated as, to the best of our knowledge, no radiative transfer code, in free access, are available to simulate optical properties of such complex particles.

Concerning *Q*_FSC_, aggregates display higher values than their SEHS. On average, aggregates are 1.35 times higher than SEHS. Moreover, we observe that *Q*_FSC_ is greatly impacted by the configuration of the aggregate and by its diameter [61]. Concerning *Q*_SSC_, values for aggregates are always smaller than for SEHS except for *C*_2_ where the SEHS’s value is equivalent. Values for aggregates are, on average, 0.81 times smaller than SEHS. We note that *Q*_SSC_ seems less sensible to the size and the type of aggregate than *Q*_FSC_. Indeed, the coefficient of variation is of 55% for *Q*_FSC_ and 34% for *Q*_SSC_. From these observations, we deduce that *Q*_SSC_ of an aggregate composed of multi-layered spheres should be lower than the efficiencies of its surface-equivalent multi-layered sphere. It results that the aggregate formation could partly explain the lower sideward efficiency of CHLAM. We note that THAL formed chains from day 9. The chains can be assimilated to the cluster *C*_2_ and *C*_3a_. Thus, this aggregations could increase the *Q*_FSC_ but can not explain the inter- and intra-species variations.

*Q*_FSC_ and *Q*_SSC_ differences between surface equivalent multi-layered or homogeneous spheres and aggregates are explained by two reasons. Firstly, the packaging involves that the scattering pattern is the result of a coherent interaction between the scattered wave emanating from each individual cell [59]. Therefore, constructive and destructive interference may occur and impact the scattering [62]. Secondly, the porosity (the void fraction) of an aggregate increases with its size. The result is that the geometrical cross section, and thus the size, are higher for an aggregate than for a sphere of same density [59]. This could explain the higher forward efficiency for aggregate as compared to their SEHS.

#### 3.4.5 Impact of the thickness of the frustule

**Fig 10:**
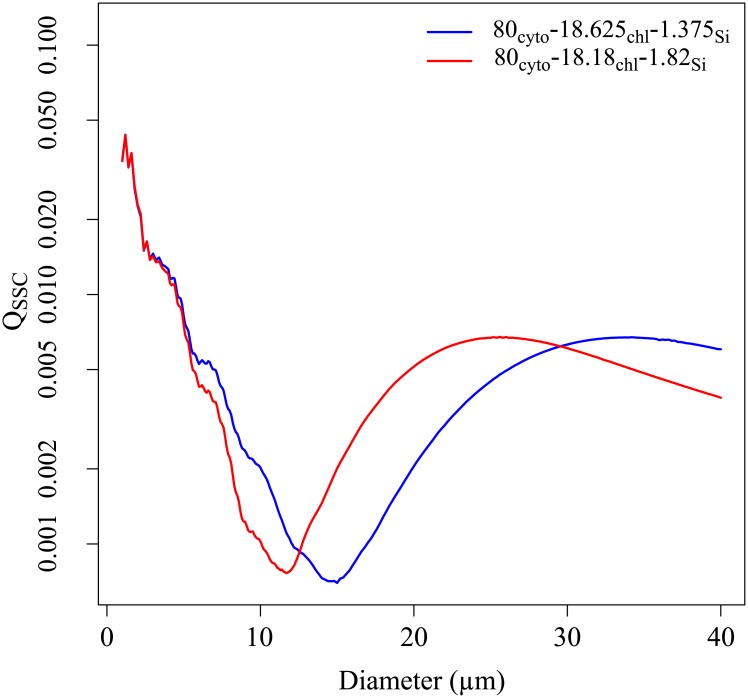
The sideward efficiency against the diameter for two models: 80%_cyt_-18.625%_chl_-1.375%_Si_ (blue lines) and 80%_cyt_-18.18%_chl_-1.82%_Si_ (red lines) and for a complex refractive index of the chloroplast (*m*(chl)) of 1.1237 + *i*0.0520.

Variations of the thickness of the frustule were observed from SEM micrographs taken on days 5, 11, 13 and 17 of the experiment (Fig 4). The proportion of the thickness of the silica wall ranged within 1.09% and 1.82% of the total cell volume. Radiative transfer computations showed that variations of the thickness frustule affected mainly the sideward scattering [11]. From days 5 to 13, the proportion of the thickness of the frustule increased from 1.37% to 1.82%. In the following, we will study the potential link between the frustule thickness and ${\tilde{Q}}_{\text{SSC}}$. Radiative transfer simulations were performed with the ScattnLay code. They were focused on the analysis of the impact of an increase of the thickness of the silica wall on the scattering intensity. For this purpose, we simulated the optical properties of three-layered sphere models. The relative volume of the frustule was 1.375% and 1.82% as measured from SEM. As the relative volumes of the cytoplasm and chloroplast cannot be measured by SEM, we considered a mean *V*_cyt_ of 80% and *V*_chl_ is calculated as the remaining volume. It results that the relative volumes of cytoplasm/chloroplast/silica wall were 80%_cyt_-18.63%_chl_-1.37%_Si_ and 80%_cyt_-18.2%_chl_-1.8%_Si_. Fig 10 displays Q_SSC_ against the cell diameter considering a complex refractive index of the chloroplast layer (*m*(chl)) of 1.1237 + *i*0.0520. The corresponding volume equivalent complex refractive indices are given in Table 2 for the two models. The silica wall may impact the sideward efficiency in two ways. First, due to some reflection on its outside surface, the silica wall reduced the penetration of light inside the cell and thus reduced the number of photons to be scattered. Second, the light inside the cell undergoes total reflection onto the inside surface of the silica wall, increasing the chance for a photon to be absorbed by the pigments [4]. This can explain why the behavior of Q_SSC_ against the diameter differs from the one observed for two-layered spheres (Fig 8). Concerning the three-layered sphere models (Fig 10), Q_SSC_ values are in the same order of magnitude whatever the thickness of the silica wall is. Other radiative transfer computations were carried out considering other *m*(chl) values but they gave similar results. We concluded that the temporal course of *Q*_SSC_ could not be explained by the variations of the silica wall thickness.

**Table 2:** Complex refractive index of the chloroplast and the volume equivalent complex refractive index for the 80%_cyt_-18.625%_chl_-1.375%_Si_ and the 80%_cyt_-18.18%_chl_-1.82%_Si_ models.

		80%_cyt_-18.625%_chl_-1.375%_Si_	80%_cyt_-18.18%_chl_-1.82%_Si_
Chl^1^	*m*_r_(chl)	1.1237	1.1237
	*m*_i_(chl)	0.0520	0.0520
EVS^2^	*m*_r_	1.04	1.0398
	*m*_i_	0.01	0.01

^1^ Chloroplast

^2^ Equivalent refractive index

### 3.5 Optical properties as a function of the growth phase

In section 3.2, three growth phases were identified for each species. For THAL, the three phases corresponded to an exponential phase (day 1 to 5), a senescent phase (day 6 to 12) and a stationary phase (day 13 to 20). For CHLAM, the Fig 3 displays an exponential phase (day 1 to 9), a stationary phase (day 10 to 15) and a phase characterized by a succession of increase and decrease in the abundance of living phytoplankton cells (day 16 to 20). The different phases are named 1, 2 and 3 for THAL and A, B and C for CHLAM. The Table 3 presents the ${\tilde{Q}}_{\text{FSC}}$, ${\tilde{Q}}_{\text{SSC}}$, ${\tilde{Q}}_{{\text{b}}_{\text{b}}}$, ${\tilde{Q}}_{{\text{a}}_{\text{phy}}}$, $\tilde{D}$ and the $\text{Chl-}a.{\text{cell}}_{\text{liv}}^{-1}$ averaged over each phase of THAL and CHLAM.

**Table 3:** The ${\tilde{Q}}_{\text{FSC}}$, ${\tilde{Q}}_{\text{SSC}}$, ${\tilde{Q}}_{{\text{b}}_{\text{b}}}$, ${\tilde{Q}}_{{\text{a}}_{\text{phy}}}$, $\tilde{D}$ and $\text{Chl-}a.{\text{cell}}_{\text{liv}}^{-1}$ values, averaged over the different population growth stages of THAL and CHLAM with their associated coefficients of variation (%).

		Phases		
		1	2	3
THAL	$<{\tilde{Q}}_{\text{FSC}}>$	7.29×10^−1^ (37)	8.09×10^−1^ (21)	6.97×10^−1^ (17)
	$<{\tilde{Q}}_{\text{SSC}}>$	5.51×10^−3^ (25)	9.05×10^−3^ (34)	8.16×10^−3^ (14)
	$<{\tilde{Q}}_{{\text{b}}_{\text{b}}}>$	1.18×10^−3^ (24)	2.15×10^−3^ (39)	2.02×10^−3^ (16)
	$<{\tilde{Q}}_{{\text{a}}_{\text{phy}}}>$	3×10^−1^ (54)	3.59×10^−1^ (43)	5.65×10^−1^ (62)
	$<\tilde{D}>(\mu m)$	6.45 (25)	8.38 (26)	10.06 (22)
	$<\text{Chl-}a.{\text{cell}}_{\text{liv}}^{-1}>(mg.m^{-3})$	5.79×10^−10^ (90)	1.77×10^−9^ (53)	7.4×10^−9^ (46)
		Phases		
		A	B	C
CHLAM	$<{\tilde{Q}}_{\text{FSC}}>$	4.70×10^−1^ (36)	2.72×10^−1^ (19)	2.82×10^−1^ (08)
	$<{\tilde{Q}}_{\text{SSC}}>$	3.9×10^−3^ (16)	5.08×10^−3^ (15)	5.13×10^−3^ (05)
	$<{\tilde{Q}}_{{\text{b}}_{\text{b}}}>$	8.42×10^−4^ (17)	1.14×10^−3^ (17)	1.15×10^−3^ (05)
	$<{\tilde{Q}}_{{\text{a}}_{\text{phy}}}>$	7.46×10^−1^ (48)	1.26 (45)	1.52 (46)
	$<\tilde{D}>(\mu m)$	7.69 (20)	8.34 (05)	8.15 (02)
	$<\text{Chl-}a.{\text{cell}}_{\text{liv}}^{-1}>(mg.m^{-3})$	4.53×10^−9^ (56)	9.82×10^−9^ (27)	8.14×10^−9^ (13)

Concerning the inter-species variations, the scattering efficiencies were always lower for CHLAM than for THAL, whatever the phase. This was due to the higher absorption of CHLAM, which is linked to the higher $<\text{Chl-}a.{\text{cell}}_{\text{liv}}^{-1}>$ content (see section 3.4.3). Indeed, the Table 3 shows that $<\text{Chl-}a.{\text{cell}}_{\text{liv}}^{-1}>$ and $<{\tilde{Q}}_{{\text{a}}_{\text{phy}}}>$ were always greater for CHLAM than for THAL, whatever the phase. Furthermore, as discussed in the section 3.4.3, the lower scattering efficiencies of CHLAM can be explained by a lower refractive index due to smaller *V*_*chl*_, which induced lower pigment densities. The section 3.4.4 showed that aggregate formation can also contribute to lower scattering efficiencies.

For THAL and CHLAM, the intra-species variations of $<{\tilde{Q}}_{\text{FSC}}>$ are negatively correlated with the cell diameter but this effect can be greatly counterbalanced by the absorption efficiency cells and/or aggregation. For THAL between the phase 1–2 and for CHLAM between the phase A-B, the $<{\tilde{Q}}_{{\text{a}}_{\text{phy}}}>$ increases with the increase of $<\text{Chl-}a.{\text{cell}}_{\text{liv}}^{-1}>$ but it did not reduce the sideward efficiencies. It shows that as demonstrated in the section 3.4.3, the evolution of $<{\tilde{Q}}_{\text{SSC}}>$ and thus $<{\tilde{Q}}_{{\text{b}}_{\text{b}}}>$ were mainly explained by the increase of the refractive index of the chloroplast layer due to higher pigment densities induced by the increase of $<\text{Chl-}a.{\text{cell}}_{\text{liv}}^{-1}>$. For CHLAM, we considered that no significant changes occurs between the phase B a C as all the parameters remain relatively constant. For THAL, the efficiencies decreased between phases 2 and 3. These variations were probably due to a combined effect of: a higher $<{\tilde{Q}}_{{\text{a}}_{\text{phy}}}>$, a higher proportion of aggregated cells and an increase of the cell diameters. First, during the phase 2 $<{\tilde{Q}}_{{\text{a}}_{\text{phy}}}>$ was 1.57 times higher than during the phase 3. Second, aggregates represented, on average, 23.6% of the total number of phytoplankton cells during the phase 2 while they represented about 39.7% of the total number of phytoplankton cells during the phase 3. This increase in the number of aggregate could reduce the sideward efficiencies (see section 3.4.4). Finally, as showed in the Figs 8 and 10, an increase of the cell diameters may lead to a decrease of the efficiencies.

## 4 Summary and remarks

In many studies, laboratory measurements of phytoplankton scattering properties were performed exclusively during the exponential growth phase to avoid the presence of detritus as phytoplankton scattering is derived from measurements of the bulk scattering and the particle size distribution. The present study shows that the use of analytical flow cytometry can avoid this issue and to allow the analysis of the evolution of the scattering properties of phytoplankton over the various phases of a culture. The temporal course of Q_FSC_ and Q_SSC_ were analyzed during the entire life-cycle of two phytoplankton species: the diatom *Thalassiosira pseudonana* (THAL) and the green algae *Chlamydomonas concordia* (CHLAM). Over the whole experiment, the forward and sideward efficiencies of THAL were on average 2.2 and 1.6 times higher than for CHLAM, respectively. We also observed intra-species variations of the forward, sideward and thus, backward efficiencies which differ significantly according to the population growth stage of the considered species. Such an observation would obviously need to be re-examined from scattering measurements performed over a larger number of phytoplankton species. Nevertheless, it tends to show that restricting the laboratory measurements to the exponential growth phase, to avoid the presence of detritus, and then assigning a mean scattering efficiency by species does not allow a complete characterisation of phytoplanktonic scattering over its life cycle. The phytoplanktonic scattering should be described by different values of cross section or the efficiency factor according to its population growth stage. It is challenging to link quantitatively the cell characteristics with the inter- and intra-species variations using radiative transfer codes. Indeed, it is difficult to define rigorously, for each cell, the input parameters to inverse the recorded signal. The configurations of aggregates, the thickness of a silica wall (in diatoms), the occurrence and size of gas vacuole(s), the size and shape of the chloroplast and the cytoplasm and their respective complex refractive index, are measurable from different techniques (e.g., SEM, transmission electron microscopy, phase contrast), which require sometimes complex, expensive and time-consuming analyses. Moreover, to our knowledge, no code exists in free access to perform a simulation considering all of the cell characteristics together. This is the reason why the radiative transfer codes were used in this study just to identify which variable(s) drive(s) the scattering and not to accurately inverse the recorded signals. The forward efficiency is impacted by aggregation and, as expected, by the cell size as the forward scattering is influenced by the diffraction. The silica wall of diatoms is *a priori* not at the origin of the higher sideward efficiencies observed for THAL, as theoretical simulations showed that the presence of the frustule does not significantly increase the sideward scattering. Nevertheless, the presence of a silica wall changes the behavior of the sideward efficiency against the diameter. The intra- and inter-species variations of the sideward efficiencies seem mainly due to the changes of the real refractive index of the chloroplast. As the chloroplast real refractive index is the key parameter, a simplistic two-layered model (cytoplasm-chloroplast) seems particularly appropriate to represent the phytoplankton cells. Note that Bernard *et al*., 2009 [20] obtained the same conclusions as they designated the 80%_cyt_-20%_chl_ model as the most appropriate model to reproduce *in situ* measurements of the remote-sensing reflectance during a phytoplankton bloom.

The next steps involve deriving the scattering cross sections and efficiencies factors of living phytoplankton cells in coastal and open waters. It will be interesting to investigate whether inter-species variations can easily be identified for the different competitive phytoplankton species coexisting in the natural environment. The species chosen for this study were morphologically distinct, which may not be the case in natural water. In addition, it will be interesting to study if significant intra-species variations will be observed in natural waters, for example, during the different phase of a phytoplankton bloom.
